# Artificial homeostatic temperature regulation via bio-inspired feedback mechanisms

**DOI:** 10.1038/s41598-023-31963-4

**Published:** 2023-03-27

**Authors:** Petro Feketa, Tom Birkoben, Maximiliane Noll, Alexander Schaum, Thomas Meurer, Hermann Kohlstedt

**Affiliations:** 1 Chair of Automation and Control, Kiel University, Kaiserstraße 2, 24143 Kiel, Germany; 2 Chair of Nanoelectronics, Kiel University, Kaiserstraße 2, 24143 Kiel, Germany; 3 Digital Process Engineering Group, Institute of Mechanical Process Engineering and Mechanics, Karlsruhe Institute of Technology (KIT), Kaiserstraße 12, 76131 Karlsruhe, Germany; 4 Kiel Nano, Surface and Interface Science KiNSIS, Kiel University, Christian-Albrechts-Platz 4, 24118 Kiel, Germany; 5 Present Address: School of Mathematics and Statistics, Victoria University of Wellington, PO Box 600, 6140 Wellington, New Zealand

**Keywords:** Electrical and electronic engineering, Applied mathematics

## Abstract

Homeostasis comprises one of the main features of living organisms that enables their robust functioning by adapting to environmental changes. In particular, thermoregulation, as an instance of homeostatic behavior, allows mammals to maintain stable internal temperature with tightly controlled self-regulation independent of external temperatures. This is made by a proper reaction of the thermoeffectors (like skin blood vessels, brown adipose tissue (BAT), etc.) on a wide range of temperature perturbations that reflect themselves in the thermosensitive neurons’ activity. This activity is being delivered to the respective actuation points and translated into thermoeffectors’ actions, which bring the temperature of the organism to the desired level, called a set-point. However, it is still an open question whether these mechanisms can be implemented in an analog electronic device: both on a system theoretical and a hardware level. In this paper, we transfer this control loop into a real electric circuit by designing an analog electronic device for temperature regulation that works following bio-inspired principles. In particular, we construct a simplified single-effector regulation system and show how spiking trains of thermosensitive artificial neurons can be processed to realize an efficient feedback mechanism for the stabilization of the a priori unknown but system-inherent set-point. We also demonstrate that particular values of the set-point and its stability properties result from the interplay between the feedback control gain and activity patterns of thermosensitive artificial neurons, for which, on the one hand, the neuronal interconnections are generally not necessary. On the other hand, we show that such connections can be beneficial for the set-point regulation and hypothesize that the synaptic plasticity in real thermosensitive neuronal ensembles can play a role of an additional control layer empowering the robustness of thermoregulation. The electronic realization of temperature regulation proposed in this paper might be of interest for neuromorphic circuits which are bioinspired by taking the basal principle of homeostasis on board. In this way, a fundamental building block of life would be transferred to electronics and become a milestone for the future of neuromorphic engineering.

## Introduction

**Figure 1:**
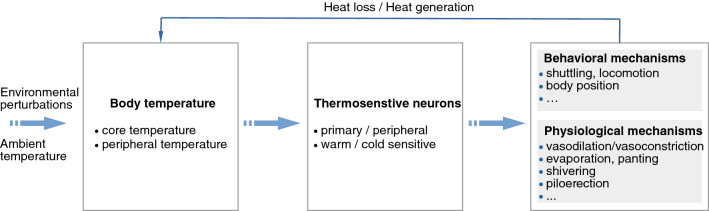
A scheme of homeostatic thermoregulation.

The biological term *homeostasis* introduced by Cannon^1^ refers to an organism’s ability to maintain steady states of operation while adjusting to changing external conditions^2–4^. In particular, thermoregulation, as an instance of homeostatic behavior, allows homeotherms, e.g., warm-blooded animals, such as birds and mammals, to maintain stable internal temperature with tightly controlled self-regulation independent of external (ambient) temperatures^5^. Ambient temperature deviations serve as perturbation sources for the organism’s core and peripheral temperatures whose states are monitored by widespread assemblies of thermosensitive neurons. Their signals go through dedicated neuronal pathways and integration centres, and can activate a plethora of different behavioral and physiological thermoregulation mechanisms (see Fig. 1). It is worth mentioning that the available thermoregulation mechanisms and their effect on the body temperature are orchestrated and coordinated not only through neuronal and endocrine systems but also through the internal temperature as the entity shared by all components involved in homeostatic thermoregulation^6,7^. Moreover, as it is pointed out in^8^, many different homeostatic systems can operate simultaneously and in general interact. Their interplay and cross-influence give rise to the regulation of complex high-level processes.

Being a hallmark of central organizing principle of physiology^4^, homeostasis increasingly attracts researchers aiming at the development of artificial systems for the purposes of bio-inspired electronics^2,9^, neuromorphic engineering^10–12^, soft robotics^13^, and control systems engineering^14–16^. It is expected that mimicking basal functional operation of living organisms, like homeostasis, and transferring their principles to artificial systems will lead to new flexible and robust control design architectures^16^ and efficient neuromorphic computing applications^12^.

The pioneering approaches for the system theoretic description of homeostatic regulation were based on the concept of reference signal that provides a sort of a standard to which a homeostatic system attempts to conform^17^. The necessary component in this case is the so-called *comparator* that compares the reference signal to the actual one and feeds back the difference of these signals through available actuation mechanisms. Such an approach has been later questioned, especially in the context of homeostatic thermoregulation^18^ since the term *temperature set-point* as an analog to the reference signal does not find its realization in actual thermoregulation systems of living organisms. It is claimed that the set-point arises from the balance of the heat loss and heat supply, and the thermoregulation system is seen as a distributed multi-sensor multi-effector adaptive system with proportional feedback control^18^. In our study we adopt the above approach and experimentally show how the set-point emerges from the balance of environmental disturbances and cold- and heat defensive counteractions of the system in an abstracted single-actuator case. To this end, we construct an electric circuit that realizes an abstracted single-effector regulation system and show how spike trains of thermosensitive artificial neurons implemented as relaxation oscillators can be processed to provide an efficient and robust feedback mechanism for the stabilization of the a priori unknown but system-inherent set-point. We additionally provide a theoretical assessment of the robust stability property for the set-point within the input-to-state stability (ISS) framework^19^. Although the coupling connections between thermosensitive artificial neurons are generally not necessary, we show that such links can be beneficial for the set-point regulation via adjustments of coupling strengths, and, therefore, we hypothesize that the synaptic plasticity in real thermosensitive neuronal ensembles can play a similar role.

A distinctive feature of our approach is the use of spike trains as information carrier rather than conventional continuous signals. A utilization of spiking neuronal-like signals for control purposes dates back to the work of DeWeerth et. al.^20^ who provided theoretical justification and experimental validation of spiking control of an electrical motor. In contrast to digital computers, the proposed event-based analog control paradigm benefits from the high temporal resolution of events that is not restricted by a digital clock, and from the amplitude resolution of thresholds that is not restricted by bits. Instead, the temporal and amplitude resolutions of the event thresholds are adaptive through classical averaging and ensemble mechanisms^21^. Additionally, spiky neuronal signals may not only enhance the efficiency and robustness of the existing conventional digital control strategies, but also pave a path to a common communication language for the human-machine interaction and accelerate the development of neuro-robotic applications.

## Results

**Figure 2:**
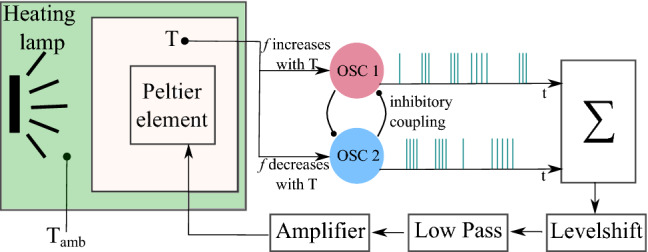
Functional diagram of the thermoregulation circuit. The oscillators OSC 1 and OSC 2 change their frequency *f* in dependence of the temperature T and inhibit each other. One oscillator charges and the other one discharges the output capacitor that integrates the spikes into an output voltage. This output voltage is level shifted, low pass filtered and amplified before it is fed into a Peltier element. The Peltier element is able to cool or to heat up in dependence of the input signal. The temperature of the Peltier element influences the temperature dependent thermistors in the oscillators and therefor the oscillator frequencies. The ambient temperature $T_{{A}mb}$ perturbs the temperature T of the system. Here a heating lamp is used to achieve a strong impact.

In the following, the general setup is first presented in an abstract way together with its robust stability assessment. It is then translated into a specific electronic circuit, and particular experimental scenarios are detailed and the obtained results are discussed. The general setup is shown in Fig. 2 in form of an abstracted functional diagram of the considered thermoregulation circuit. Two temperature-sensitive oscillators (OSC 1 and OSC 2) are connected to a base plate with a temperature *T* that is influenced by a Peltier element. The oscillators change their frequencies in opposite ways in response to temperature changes, i.e., while one frequency increases the other one decreases. In addition both oscillators inhibit each other. The generated spikes influence the processing unit $\Sigma$ in opposite ways, i.e., one achieves an increase in its internal state while the other one implies a decrease. The net effect produces a level shift for the actuator signal which is low pass filtered and amplified to produce the input for the Peltier element. Here, a heating lamp is used to increase the ambient temperature $T_{{A}mb}$ and thus serves as a perturbation source for the base plate’s temperature *T*.

### Abstract mathematical model

Let $T(t)\in {\mathbb {R}}$ and $u(t)\in {\mathbb {R}}$ denote the inner temperature and the integrated signal *u* at time $t\ge 0$, i.e., the output voltage of the level shift, respectively. Then, their dynamics can be modeled as

1a $$\begin{aligned} \dot{T}&= \alpha (T_{Amb}(t)-T) - F(u), \end{aligned}$$

1b $$\begin{aligned} \dot{u}&= -\gamma u + r_{H}(t) - r_{C}(t), \end{aligned}$$

where $r_{H}(t)>0$ and $r_{C}(t)>0$ stand for the spike trains of the warm- and cold-sensitive artificial neurons, respectively, $T_{Amb}(t)\in {\mathbb {R}}$ denotes the ambient temperature that serves as a source for the temperature perturbations, $F:{\mathbb {R}} \rightarrow {\mathbb {R}}$ is the feedback gain function with $F(0)=0$, $\gamma >0$ and $\alpha >0$ are positive parameters. In sequel, we assume that the dynamics of thermosensitive artificial neurons is much faster than the energy interchange between the plate and the environment. This implies that there exists a function $h:{\mathbb {R}} \rightarrow {\mathbb {R}}$ such that $r_{H}(t) - r_{C}(t) = h(T(t))$, i.e., at every moment of time *t* the influence of spike trains on variable *u* depends only on the internal temperature *T*. This means that the temperature set-point $T_{Set}$ corresponds to the zeros of function *h*, i.e., thermosensitive artificial neurons do not produce any signal for actuators to warm up or cool down when *h* is zero, and support cooling/heating when *h* is nonzero. From the above discussion it is clear that by changing the parameters of neurons one changes the properties (zeros) of function *h* and, therefore, regulates the set-point $T_{{S}et}$. In our experimental setup, such regulation has been achieved by adjusting the coupling strengths and led to the decrease of the set-point from $29^\circ$ to $27^\circ$ (see Fig. 4, time interval $t \in [400\,\text {s}, 600\,\text {s}]$). At this moment it is, however, not clear if the set-point is stable and robust with respect to the temperature perturbations.

Introducing the new variable $e(t) = T(t)-T_{{S}et}$ and denoting $d(t) = T_{Amb}(t) - T_{Set}$, system (1) can be rewritten as

2a $$\begin{aligned} \dot{e}&= -\alpha e - F(u) + \alpha d, \end{aligned}$$

2b $$\begin{aligned} \dot{u}&= -\gamma u + {\tilde{h}}(e) \end{aligned}$$

with the state $(e(t),u(t))^\top \in {\mathbb {R}} \times {\mathbb {R}}$ and the external perturbation $d(t) \in {\mathbb {R}}$, where $T_{Set} \in {\mathbb {R}}$ denotes the set-point, i.e., the temperature that turns *h* to zero, and ${\tilde{h}}(e) := h(T_{{S}et}+e)$ with $\tilde{h}(0)=0$. The newly introduced quantities *e* an *d* can be interpreted as the deviation of the real temperature *T* from the set-point $T_{Set}$, and the environmental perturbation of the set-point, respectively. In sequel, we aim to derive sufficient conditions for the input-to-state stability of system (2). This property guarantees that the temperature deviation *e* will eventually converge to the $\delta (\left\| d\right\| _\infty )$-neighbourhood of zero, where function $\delta \in {\mathcal {K}}$ is the ISS-gain from equation (4) (please see “Methods” section for details).

### Robust stability assessment

Let us consider system (2) assuming both *F* and ${\tilde{h}}$ to be monotone and globally Lipschitz continuous functions with Lipschitz constants $F_0>0$ and $h_0>0$, respectively. Taking candidate ISS-Lyapunov function $V(e,u) = |e| + |u| > 0$, for all $(e,u)\not = 0$ we get

$$\begin{aligned} \dot{V}(e,u)&= \text {sign}(e) (-\alpha e - F(u) + \alpha d) + \text {sign}(u) (-\gamma u + {\tilde{h}}(e)) \\&= -\alpha |e| \underbrace{- \text {sign}(e)F(u)}_{\le F_0|u|} + \underbrace{\text {sign}(e)\alpha d}_{\le \alpha |d|} - \gamma |u| + \underbrace{\text {sign}(u) {\tilde{h}}(e)}_{\le h_0 |e|} \\&\le - (\alpha -h_0)|e| - (\gamma -F_0)|u| + \alpha |d| \\&\le - \xi V(e,u) + \alpha |d|, \end{aligned}$$

where $\xi = \text {min}\{\alpha -h_0, \gamma -F_0\}>0$ provided that $h_0<\alpha$ and $F_0<\gamma$. Finally,

$$\begin{aligned} \dot{V}(e,u)&\le - (1-\varepsilon ) \xi V(e,u) \quad \text {for any} \quad \varepsilon \in (0,1) \end{aligned}$$

provided that $V(e,u) \ge \frac{\alpha }{\varepsilon \xi } |d|$. Following^19^, system (2) is ISS and the proposed feedback mechanism provides the robust stabilization of temperature *T* around the set-point $T_{Set}$. Free parameter $\varepsilon \in (0,1)$ and Lipschitz constants $h_0, F_0$ can be used to tune the ISS-gain, i.e., how close *T* will converge to $T_{Set}$. Under additional assumptions on system dynamics, the ISS-gain can be even smaller. In particular, let the dynamics of *u* from (2b) be significantly faster than the temperature dynamics. In this case, $u(t) \approx \gamma ^{-1}{\tilde{h}}(e(t))$ for any *t*, and equation (2a) reads as

$$\begin{aligned} \dot{e} = -\alpha e - {\tilde{F}}(e) + \alpha d, \end{aligned}$$

where ${\tilde{F}}(e) = F(\gamma ^{-1}{\tilde{h}}(e))$ is monotone and globally Lipschitz continuous functions with Lipschitz constants ${\tilde{F}}_0>0$. Taking candidate ISS-Lyapunov function ${\tilde{V}}(e) = |e| > 0$, for all $e\not = 0$ we get

$$\begin{aligned} \dot{{\tilde{V}}}(e)&= \text {sign}(e) (-\alpha e - {\tilde{F}}(e) + \alpha d) \\&\le -(\alpha +{\tilde{F}}_0) |e| + \alpha |d|\\&= -(1-\varepsilon )(\alpha +{\tilde{F}}_0) V(e) \quad \text {for any} \quad \varepsilon \in (0,1) \end{aligned}$$

provided that ${\tilde{V}}(e) \ge \frac{\alpha }{\varepsilon (\alpha +{\tilde{F}}_0)}|d|$. The latter implies ISS from *d* to *e* and guarantees the convergence of the temperature difference *e* to the vicinity of zero, whose size can be reduced by choosing sufficiently large ${\tilde{F}}_0$. The latter assertion will be later validated experimentally in Experiment III.

The proven ISS property suggests that the temperature deviation *e* converges to a neighbourhood of zero. This, in particular, means that the temperature *T* converges close to the set-point $T_{Set}$, and the activity of both warm- and cold-sensitive neurons exhibits a certain frequency pattern that corresponds to the approximate balance of heating/cooling actuation and environmental temperature perturbations. Such patterns have been numerically investigated in^22^ and used for the reconstruction of the unknown ambient temperature using reservoir computing techniques. In case of perfectly identical parameters of oscillators (except for cold/warm sensitivity) the mentioned frequency pattern resembles frequency synchronization of oscillators in absence of external temperature perturbations.

### Device

**Figure 3:**
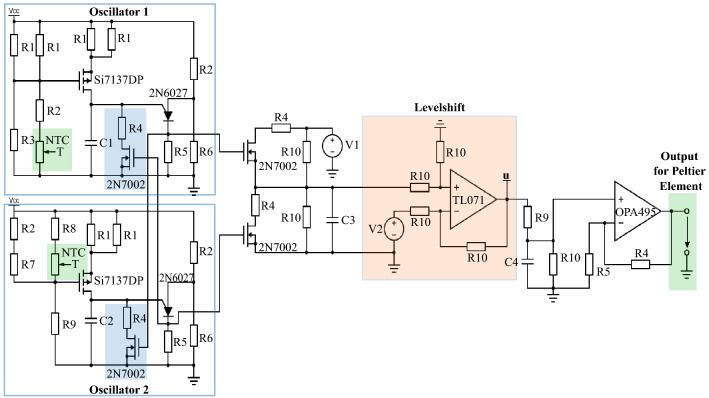
Electrical circuit of the thermoregulation. The inhibitory couplings between the oscillators are marked in solid blue. The oscillators charge/discharge a capacitor whose voltage is the input for the level shift (marked in light orange). The level shift controls the set-point of the regulation and its output voltage is called *u*. Afterwards the signal is low pass filtered and amplified. The resulting voltage is fed to a Peltier element which heats or cools the temperature dependent NTC thermistors (marked in light green) in the oscillators. All circuit parameters are listed in a table in the experimental part of the “Methods” section.

The relaxation-type oscillator is the basic building block of the device (schematically shown in Fig. 3) that reacts to changes in the ambient temperature. Here we use two relaxation-type oscillators with different charging paths. For both oscillators a voltage divider equipped with negative temperature coefficient (NTC) thermistors are used to charge the capacitors *C*1 and *C*2.

Due to the different placement of these thermistors in the voltage divider a temperature rise leads to opposite effects in the oscillators: With an increased temperature the resistances of the thermistors decrease. In oscillator 1 this leads to an increased voltage drop at the resistances *R*1. Therefore the gate-source-voltage of the p-MOSFET Si7137DP in oscillator 1 increases as well as the source-drain current. The source-drain current charges the capacitor *C*1 faster and leads to a higher frequency of oscillator 1. In oscillator 2 the gate-source-voltage of the p-MOSFET Si7137DP decreases with increasing temperature. Therefore capacitor *C*2 is charged slower and the frequency of oscillator 2 decreases. As a consequence a temperature change implies a change in the frequency difference between both oscillators. Furthermore the oscillators inhibit each other equally (marked in solid blue boxes in Fig. 3). When oscillator 1 generates a voltage spike at the cathode of the 2N6027 this voltage is used to switch on an n-MOSFET that discharges the capacitor *C*2 of the oscillator 2 for the duration of the spike. When oscillator 2 spikes the same mechanism is used to discharge the capacitor *C*1 of oscillator 1. The generated output spikes of both oscillators are used to charge (oscillator 1) and discharge (oscillator 2) the capacitor *C*3 and are therefore added up, or subtracted, respectively, in the capacitor voltage. This voltage is fed into an operational amplifier used as level shift. The level shift increases or decreases the voltage in dependence of its input voltage *V*2. Parameter *V*2 (along with other parameters, e.g., of oscillators) influences the particular value of the set-point. However, the exact relation between the *V*2 (or any other parameters) and the resulting value of the set-point is unknown in advance, that is why we call the set-point system-inherent. In order to construct our device, we need this system-inherent set-point to be within a reasonable distance to the ambient temperature and the initial temperature of the plate. For this reason, we fix all parameters of the system (e.g., oscillator’s parameters, coupling strength, etc.) and tune the level shift voltage *V*2 so that the resulting set-point is close to $T_{Amb}$ (herein no additional temperature perturbation using a lamp is applied). With this we arrive to the value $V2= 0.5 V$ and use it for all experiments. The output voltage *u* is low pass filtered to avoid abrupt changes in the voltage the Peltier element receives. The low pass filtered voltage is then amplified before it is used to power a Peltier element. The Peltier element has the ability to heat or to cool in dependence of the incoming voltage. The NTC thermistors of both oscillators are placed on top of the Peltier element and change their resistances depending on the actions of the Peltier elements. These resistance changes influence the respective oscillator frequencies and therefore also the output voltage that the Peltier element receives. This completes the control loop shown schematically in Fig. 2. In order to measure the ambient and the inner temperatures two more thermistors are used. One is placed on the Peltier element (to measure inner temperature *T*) and one next to the Peltier element (to measure ambient temperature $T_{{A}mb}$).

### Experimental results

**Figure 4:**
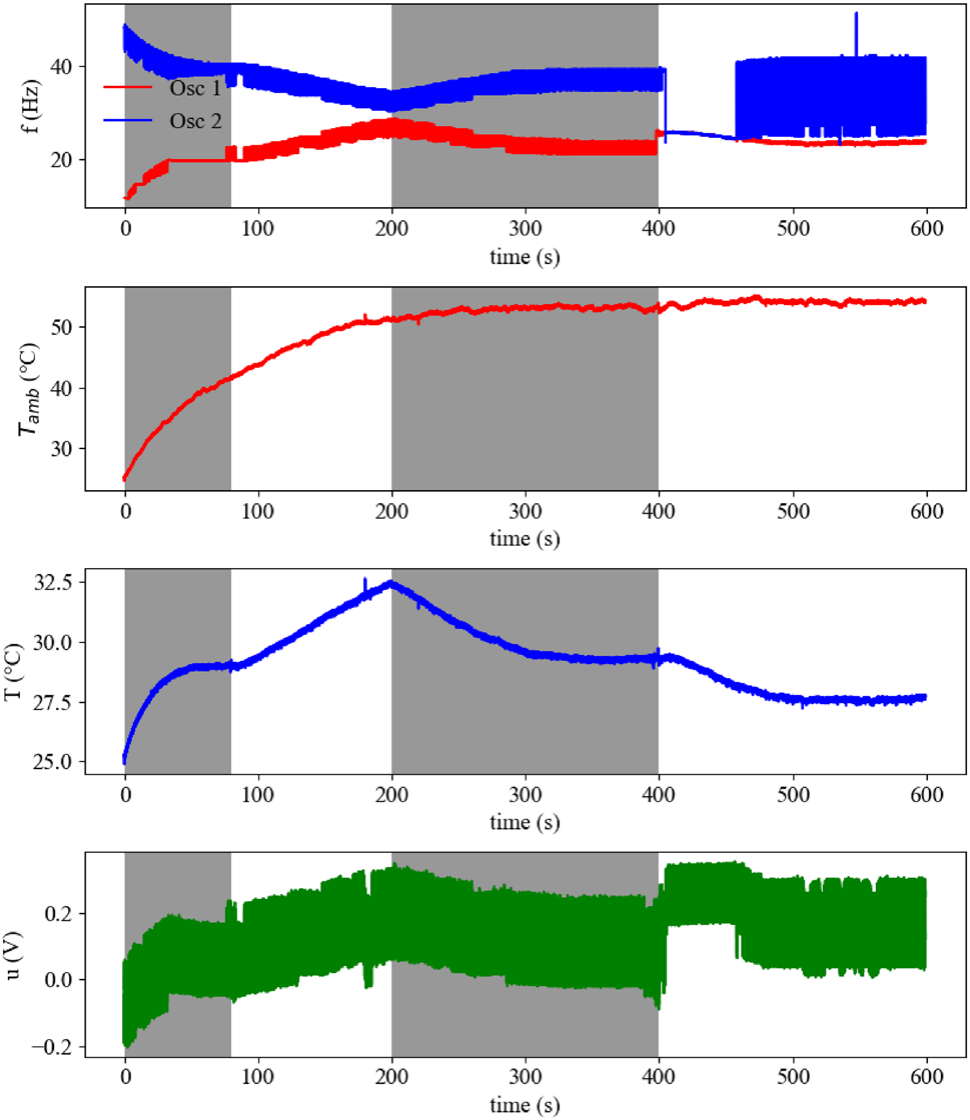
Measurement results for Experiment I. The four figures show the spiking frequency of the oscillators *f*, the ambient temperature $T_{Amb}$, the inner temperature *T* and the output voltage *u* of the level shift over time. At the start of the measurement the ambient temperature $T_{{A}mb}$ approximately equals the inner temperature *T*. A heating lamp is used as constant temperature perturbation during the experiment and turned on at the start of the measurement. At 80 s the proposed feedback mechanism is turned off for 120 s. The inhibitory weights of the oscillators are adapted at 400 s. The different parts of the measurement are separated by background color. Each the ambient and the inner temperature are displayed as values averaged over the last 10 measurement points to reduce noise.

In this subsection, we present and discuss experimental results of the device functioning under various perturbation scenarios.

*Experiment I:* At the start of the experiment (see Fig. 4) a heating lamp is turned on and heats up the area where the Peltier element with the three thermistors and the fourth thermistor are located. The inner temperature is measured by one of the thermistors on top of the Peltier element. The fourth thermistor next to the Peltier element measures the ambient temperature. This area is separated from the main part of the device. This local separation ensures that the electronic components are not influenced by the applied temperature perturbation. The device consists of the rest of the electrical circuit and the two thermistors on top of the Peltier element that are part of the charging paths in the oscillators. So the device is the complete electrical circuit shown in Fig. 3.

In the first 80 s we can see a clear influence of the device on the inner temperature. The ambient temperature has already exceeded 40 $^{\circ }$ while the inner temperature is still under 30 $^{\circ }$ and it has reached a steady-state. At 80 s the feedback mechanism is turned off and the Peltier element stops the cooling. This leads to the slow but distinct rise in the inner temperature. The rise time is lower than the one of the ambient temperature because the Peltier element is placed below a metal plate with thermally conductive paste that only slowly heats up and therefore has a lingering cooling effect on the thermistors. At 200 s the feedback is turned on again which leads to a decrease in the inner temperature to the same level as before. To show that small changes in the synaptic weights are beneficial to the set-point regulation, at 400 s the synaptic weights are changed. Before there was an equal inhibitory influence from both oscillators due to equal discharging resistances $R4 = 10\,k\Omega$. Now the resistance of oscillator 1 is increased to $100\,k\Omega$ and the one of oscillator 2 is decreased to $1\,k\Omega$. This leads to an even further reduction in the inner temperature to approximately 27 $^{\circ }$.

**Figure 5:**
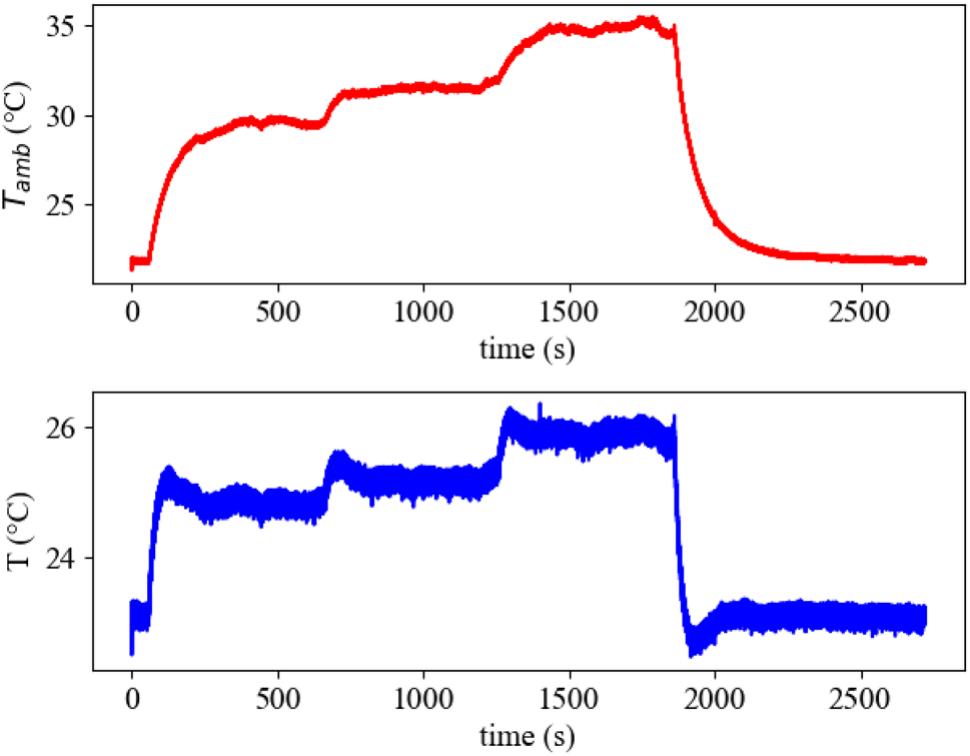
Measurement results for Experiment II. The two figures show the ambient temperature $T_{Amb}$ and the inner temperature *T* under a subset of different temperature perturbations. At the beginning the inner temperature is in vicinity of the ambient temperature. Each the ambient and the inner temperature are displayed as values averaged over the last 10 measurement points to reduce noise.

**Figure 6:**
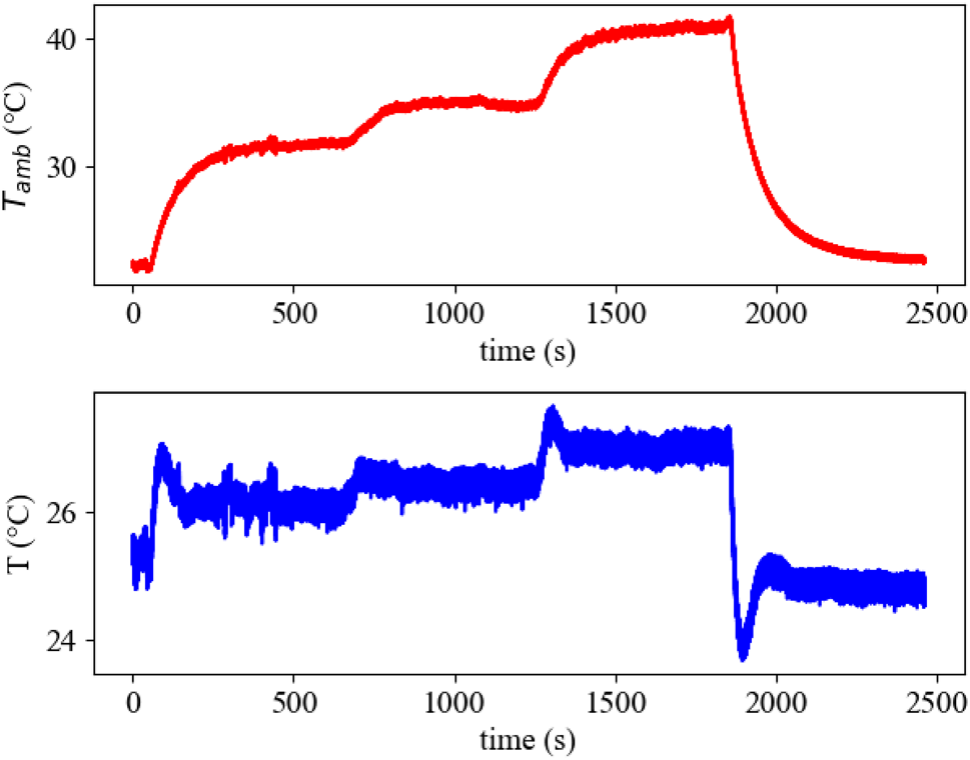
Measurement results for Experiment III. The two figures show the ambient temperature $T_{Amb}$ and the inner temperature *T* under a subset of different temperature perturbations. Here, we increased the amplifier gain of the circuit by a factor of 4.4 by increasing the $R_4$ of the amplifier from $10\ k\Omega$ to $47\ k\Omega$. This leads to a reduced deviation from the original set-point without temperature perturbation. Each the ambient and the inner temperature are displayed as values averaged over the last 10 measurement points to reduce noise.

*Experiment II:* The performance of the device under time-varying temperature perturbations is depicted in Fig. 5. At the start of the experiment the inner temperature *T* is in a vicinity of the ambient temperature $T_{Amb}$. Despite strong external temperature perturbations, the inner temperature *T* deviates from the original set point in the magnitude of ca. 3$^{\circ }$ in the course of the experiment. This deviation can be decreased by increasing the amplifier gain in the control loop that has been made in *Experiment III:* Fig. 6 showcases the performance of the device with a gain of 48 instead of 11 used in Experiment II. The deviation decreased to ca. 2 $^{\circ }$ even though the temperature perturbation used was stronger, as can be seen by the 5 $^{\circ }$ warmer ambient temperature $T_{Amb}$.

## Discussion

The electronic realization of homeostatic thermoregulation proposed in this paper addresses the questions of robust stabilization and the role of couplings between the thermosensitive artificial neurons for temperature tuning. In particular, we have shown experimentally how spike trains generated by the temperature-driven relaxation-type oscillators can be processed to realize an efficient feedback mechanism for the temperature set-point regulation and stabilization, and how the set-point can be manipulated by adjusting the coupling strengths of (inhibitory) connections between thermosensitive artificial neurons. The latter result supports the idea of *control on sensory level*, i.e., no change on the actuator side is necessary, and it is in accordance with the approaches of theoretical and experimental neuroscience^7,23^. The robustness of the proposed feedback control is also accessed theoretically within the input-to-state stability framework using an abstracted mathematical representation of the considered control loop.

It is worth mentioning that the set-point is inherently defined by the system and it is not prescribed externally. It is, however, challenging to identify this set-point in advance due to a complex dynamical dependence between the spike trains of thermosensitive artificial neurons and the energy exchange between the plate, environment, and Peltier element. To overcome this issue, we first prove the robust stability of the *unknown* set-point utilizing time scales separation technique in combination with conventional Lyapunov-based approach for stability analysis of nonlinear systems. We also show that stability and robustness properties of *different* set-points emerge from the interplay between the feedback control gain and activity patterns of thermosensitive neuronal ensembles. In the considered case of two inhibitory connected warm- and cold-sensitive oscillators, the term “activity pattern” corresponds to a certain ratio between the firing frequencies of oscillators that corresponds to the approximate balance of heating and cooling. A plethora of such patterns has been revealed in^22^ by numerical simulations of temperature-sensitive neuron models^24^ given by Hodgkin-Huxley formalism. These theoretical conclusions are in accordance with the experimental results of the paper, which demonstrate that connections between thermosensitive artificial neurons can be beneficial for the set-point regulation, and the adjustments of the coupling strength can lead to a better disturbance rejection (see Fig. 4, time interval $t \in [400\,\text {s}, 600\,\text {s}]$). We also showcase in experiments how the ISS-gain can be regulated by the feedback amplifier and how this allows to maintain the inner temperature within a narrow region around the set-point (see Figs. 5 and 6).

The electronic realization of temperature regulation proposed in the paper might be of interest for neuromorphic circuits, which are bioinspired by taking the basal principle of homeostasis on board. In this way, a fundamental building block of life would be transferred to electronics and become a milestone for the future of neuromorphic engineering.

Finally, we would like to emphasize two aspects that are of a particular interest and importance for the future research: (1) multi-effector system without direct connections between different actuation mechanisms through the nervous or endocrine system, and (2) the criticality property of ensembles of sensory neurons. Observations of thermal physiologists^6^ suggest that the coordination of different thermoeffectors is achieved not through neural connections, but through the temperature as an entity shared by all effectors. A successful utilization of this principle in artificial electric circuits may provide additional flexibility layer for distributed feedback control and additional communication path between actuators in form of physical interactions through the energy exchange. Also, the two-neuron setup considered in our paper can be replaced with a more realistic setup of large-scale distributed sensory network, and it is of interest to study the influence of the criticality property^25,26^ of the network on the robustness and performance of thermoregulation.

## Methods

We start with presenting necessary control-theoretic tools for stability analysis and robustness assessment, followed by the details of the experimental implementation, including all parameters of the components.

### Input-to-state stability of systems with external inputs

Consider a system of ordinary differential equations with external input

3 $$\begin{aligned} {\dot{x}} = f(x, d),\quad x(0) = x_0, \end{aligned}$$

where $x(t)\in {\mathbb {R}}^n$, $n\in {\mathbb {N}}$ and $d(t)\in \mathbb R^m$, $m\in {\mathbb {N}}$ denote the state and the input at time $t\ge 0$, respectively, function $f: {\mathbb {R}}^n \times \mathbb R^m\rightarrow {\mathbb {R}}^n$ is locally Lipschitz with $f(0, 0)= 0$, input $d\in {\mathcal {U}} = L_\infty ({\mathbb {R}}_{\ge 0},{\mathbb {R}}^m)$ – the space of Lebesgue measurable essentially bounded functions equipped with the norm $\left\| d\right\| _{\infty }:=\text {esssup}_{t\ge 0}|d(t)| = \inf _{D\subset {\mathbb {R}},\, \mu (D)=0} \sup _{t\in \mathbb R_{\ge 0}\setminus D}|d(t)|$, where $|\cdot |$ stands for the Euclidean norm. An absolutely continuous function $t\mapsto \phi (t,x_0,d)$ is called a solution to the problem (3) for a given initial condition $x_0 \in {\mathbb {R}}^n$ and a given input $d\in {\mathcal {U}}$ if $\phi (0,x_0,d(0)) = x_0$ and $\dot{\phi } = f(\phi ,d)$ holds almost everywhere.

To introduce the appropriate stability property we recall the standard definitions of comparison functions^27^: ${\mathcal {P}} := \{\,\gamma :{\mathbb {R}}_{\ge 0} \rightarrow {\mathbb {R}}_{\ge 0}\,| \,\gamma \text {\,is continuous}, \gamma (0)=0 \text {\,and\,} \gamma (r)>0\text {\,for\,}r>0\}$, ${\mathcal {K}} := \{\,\gamma \in {\mathcal {P}}\,| \,\gamma \text {\,is strictly increasing}\}$, ${\mathcal {K}}_{\infty } := \{\,\gamma \in {\mathcal {K}}\,| \,\gamma \text {\,is unbounded}\}$, ${\mathcal {L}} := \{\,\gamma :\mathbb R_{\ge 0} \rightarrow {\mathbb {R}}_{\ge 0}\,| \,\gamma \text {\,is continuous and strictly decreasing with},\, \lim \limits _{t\rightarrow \infty }\gamma (t)=0\}$, ${{\mathcal {K}}}{{\mathcal {L}}} := \{\,\beta :{\mathbb {R}}_{\ge 0}\times {\mathbb {R}}_{\ge 0} \rightarrow \mathbb R_{\ge 0}\,| \,\beta \text {\,is continuous},\, \beta (\cdot ,t)\in {\mathcal {K}} \text {\,for any\,} t\ge 0, \, \beta (r,\cdot )\in {\mathcal {L}} \text {\,for any\,} r>0\}$.

System (3) is called *input-to-state stable*^19^ (ISS) if there exist $\beta \in {{\mathcal {K}}}{{\mathcal {L}}}$ and $\delta \in {\mathcal {K}}$ such that for any initial value $x_0\in {\mathbb {R}}^n$ and any input $d\in {\mathcal {U}}$ the corresponding solution $x= \phi (\cdot , x_0, d)$ exists on $[0,\infty )$ and satisfies

4 $$\begin{aligned} |\phi (t, x_0, d)|\le \beta (|x_0|,t)+\delta (\left\| d\right\| _{\infty })\quad \text {for all} \quad t\ge 0. \end{aligned}$$

The ISS notion combines classical Lyapunov stability of the trivial solution and robustness with respect to the external perturbation *d*. In particular, it guarantees the convergence of solutions to a neighbourhood of the origin, whose size depends on the magnitude of the external perturbation.

### Details of the experimental implementation

**Figure 7:**
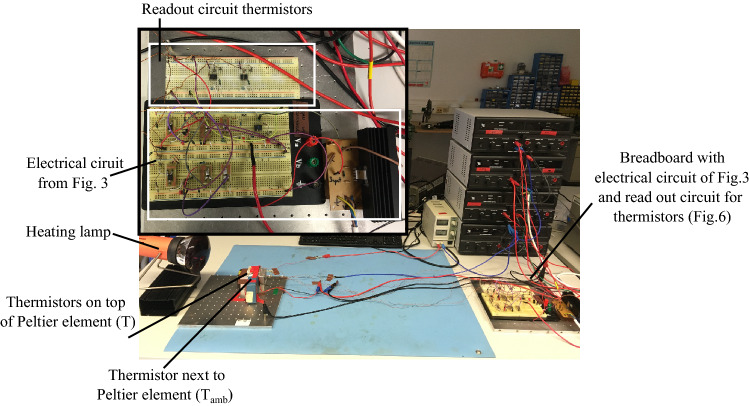
Picture of the setup. In the main picture the whole setup is shown. The circuitry on the right side is locally separated from the thermistors and the Peltier element. Only the Peltier element and the thermistors see the direct temperature perturbation gernerated with the heating lamp. In the zoomed in picture on the top left side the breadboard setup of the circuitry is shown. The breadboard on the top contains the read out circuits for the thermistors (*T* and $T_{{A}mb}$). The lower breadboard contains the electronic circuit from Fig. 3. The amplifier is located next to the breadboards.

**Figure 8:**
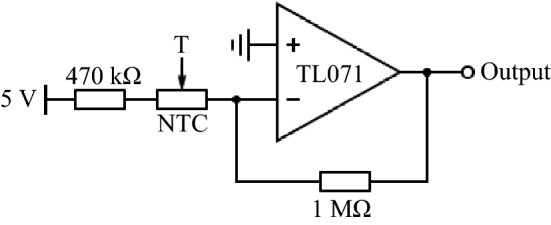
Electrical circuit to read out the ambient and inner temperature with thermistors (Vishay NTCLE1000E3, color code: orange (3 $\%$ tolerance), yellow, violet, yellow). The thermal capacitance of each thermistor is $C_{th} = \delta _{th} * \tau _c = 0.105\ \mathrm{J}\ \mathrm{K}^{-1}$. With $\delta _{th} = 7$ mW/K as dissipation factor and $\tau _c = 15$ s as thermal time constant. The measured voltage is transferred into a temperature according to the data sheet. The data sheet can be accessed via this link: https://docs.rs-online.com/ab6e/0900766b815d269a.pdf.

This subsection includes a photograph of the designed thermoregulation device (Fig. 7), a scheme of the electrical circuit to read out the ambient and inner temperature (Fig. 8), and a table summarizing all parameter values for the components used in the electronic circuit (Table 1).

**Table 1:** Parameters used for the components of the electronic circuit from Fig. 3.

Component	Value
R1	$39\ \mathrm{k} \Omega$
R2	$100\ \mathrm{k} \Omega$
R3	$470\ \mathrm{k} \Omega$
R4	$10\ \mathrm{k} \Omega$
R5	$1 \ \mathrm{k} \Omega$
R6	$200\ \mathrm{k} \Omega$
R7	$82 \ \mathrm{k} \Omega$
R8	$1 \ \Omega$
R9	$1 \ \mathrm{M} \Omega$
R10	$10\ \mathrm{M} \Omega$
C1	$0.047\ \upmu \mathrm{F}$
C2	$0.047\ \upmu \mathrm{F}$
C3	$0.047\ \upmu \mathrm{F}$
C4	$0.47\ \upmu \mathrm{F}$
$V_{CC}$	10 V
V1	$2 \ \mathrm{V}$
V2	$0.5\ \mathrm{V}$

## Data Availability

The data that support the findings of this study are available on request from the corresponding author P.F.
